# Assessment of nanoindentation in stiffness measurement of soft biomaterials: kidney, liver, spleen and uterus

**DOI:** 10.1038/s41598-020-75738-7

**Published:** 2020-11-02

**Authors:** Guanlin Wu, Michael Gotthardt, Maik Gollasch

**Affiliations:** 1 Max Delbrück Center for Molecular Medicine (MDC) in the Helmholtz Association, Robert-Rössle-Straße 10, 13125 Berlin, Germany; 2 Experimental and Clinical Research Center (ECRC), Charité–Universitätsmedizin Berlin, Berlin, Germany; 3 German Center for Cardiovascular Research (DZHK), Partner Site Berlin, Berlin, Germany; 4 Department of Internal and Geriatric Medicine, University of Greifswald, University District Hospital Wolgast, Greifswald, Germany; 5 Medical Clinic of Nephrology and Internal Intensive Care, Charité Universitätsmedizin Berlin, Berlin, Germany

**Keywords:** Nanobiotechnology, Sensors and probes

## Abstract

Nanoindentation technology with high spatial resolution and force sensitivity is widely used to measure the mechanical properties of hard biomaterials and tissues. However, its reliability to analyze soft biomaterials and organs has not been tested. Here, we evaluated the utility of nanoindentation to measure the passive mechanical properties of soft biological specimen. Kidney, liver, spleen and uterus samples were harvested from C57BL/6 N mice. We assessed test–retest repeatability in biological specimen and hydrogel controls using Bland–Altman diagrams, intraclass correlation coefficients (ICCs) and the within-subject coefficients of variation (COVs). The results were calculated using Hertzian, JKR and Oliver & Pharr models. Similar to hydrogels, Bland–Altman plots of all biological specimen showed good reliability in stiffness test and retest examinations. In gels, ICCs were larger than 0.8 and COVs were smaller than 15% in all three models. In kidney, liver, spleen and uterus, ICCs were consistently larger than 0.8 only in the Hertzian model but not in the JKR and Oliver & Pharr models. Similarly, COVs were consistently smaller than 15% in kidney, liver, spleen and uterus only in the Hertzian model but not in the other models. We conclude that nanoindentation technology is feasible in detecting the stiffness of kidney, liver, spleen and uterus. The Hertzian model is the preferred method to provide reliable results on ex vivo organ stiffness of the biological specimen under study.

## Introduction

Several diseases lead to changes in stiffness in certain organs, which could give rise to illnesses. Potentially, there is also a possibility that a variation of stiffness in tissue or cells influences the function or structure in other organs in the body. For example, patients with heart failure and a preserved ejection fraction can exhibit increases in passive myocardial stiffness^1^. Cyanotic patients with congenital heart diseases are often characterized by increased arterial stiffness in comparison with healthy population^2^. There is a direct relationship between aortic stiffness and left ventricular systolic and diastolic dysfunction in patients with inflammatory bowel disease^3^. Eradication of hepatitis C virus infection causes a significant decline in liver stiffness -particularly in patients with high baseline level of inflammation or patients who received direct-acting antiviral agents^4,5^. Increased arterial stiffness can occur in parallel with the decline of glomerular filtration rate in patients with mild-to-moderate chronic kidney disease^6^. Type 2 diabetes had greater impact on pulse wave velocity of the central arteries than peripheral arteries^7^. In clinical settings, a number of various techniques have been introduced to detect changes in stiffness of human organs, such as transient elastography, ultrasonography, acoustic radiation force impulse elastography, point shear wave elastography and magnetic resonance elastography^8–15^, which are helpful and meaningful in diagnosis of organ fibrosis. Presently, a variety of testing techniques have been developed and utilized widespread from bulk scale to the micro/nano-scale for characterizing some biomaterials *ex vivo*^16–21^. Piuma nanoindenter is one of these technologies to be used to study elastic property of biomaterials. This technique has been widely used to test passive mechanical properties of hard biosamples, such as bone and cartilage^22–26^. However, so far there are only very few studies^27–31^ which used the Nanoindentation technology to study organ stiffness of soft biomaterials, such as the kidney, liver, spleen and uterus samples. There is uncertainty on suitable ways in analyzing ex vivo organ stiffness by this technique.

## Results

### Hertzian, JKR and Oliver & Pharr models

**Figure 1:**
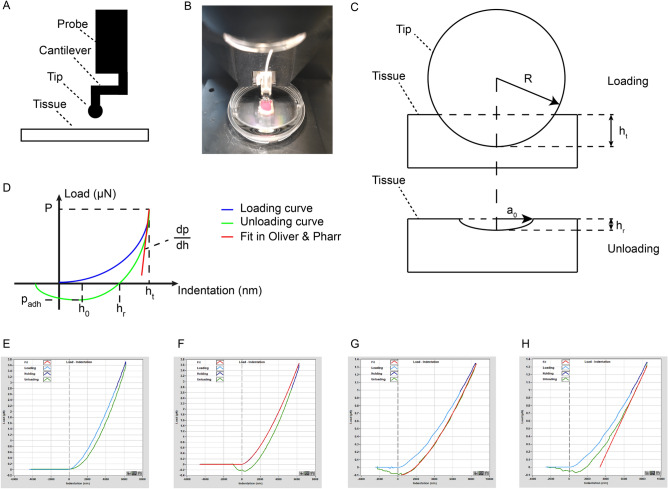
Principle of effective Young’s modulus calculation. (**A**) Details of probe and relationship between the probe and tissue. (**B**) The probe of nanoindenter was focused on an appropriate area of tissue. (**C**) Schematic diagram of indentation from loading to unloading. (**D**) Load-indentation schematic diagram. (**E**) Schematic diagram of non-adhesion indentation. (**F**) Schematic diagram of indentation in Hertzian model with 100% fit. (**G**) Schematic diagram of indentation in JKR model with 100% fit. (**H**) Schematic diagram of indentation in Oliver & Pharr model with 65–85% fit.

We applied Hertzian, JKR and Oliver & Pharr models based on the following considerations. The calculation of the effective Young’s modulus (*Eff*) by considering the Hertzian contact model^36,37^, follows the fit of the loading curve (Fig. 1F) to the following equation:

$$Eff = \frac{{P{*}3/4}}{{\sqrt R \cdot h_{t}^{3/2} }}$$

where P is the load in the peak of fit, *R* means the tip radius and *h*_*t*_ represents the indentation depth (Fig. 1C,D).

The JKR model^38^ is often used for analysis of elastic adhesive materials and could allow a better estimation of *Eff* in the presence of adhesion forces which changes the contact area (Fig. 1G). However, if the unloading part shows no sticky character, it is expected that there would be no reliable result from this model (Fig. 1E). The equation used for the fitting is:

$$h_{t} - h_{0} = \frac{{a_{0}^{2} }}{R}\left( {\frac{{1 + \sqrt {1 - \frac{P}{{P_{adh} }}} }}{2}} \right)^{\frac{4}{3}} - \frac{2}{3}\frac{{a_{0}^{2} }}{R}\left( {\frac{{1 + \sqrt {1 - \frac{P}{{P_{adh} }}} }}{2}} \right)^{\frac{1}{3}}$$

$$P_{adh} = - \frac{3}{2}\pi \Delta rR$$

$$Eff = \frac{{9\pi R^{2} \Delta r}}{{2a_{0}^{3} }}$$

where *h*_*t*_ is indentation depth, *h*_*0*_ means the contact point, a_0_ represents the contact radius at zero load, *R* is the tip radius of the indenter, *P* is the load and *P*_adh_ is the pull-off force (minimum load), $\Delta r$ means work of adhesion. *h*_*0*_ and *a*_0_ are fitting parameters **(**Fig. 1D).

For elastoplastic materials, the unloading part of the curve is often fitted by the so-called Oliver & Pharr model^39,40^, which may exclude plasticity bias. This method derives the *Eff* from the slope of the unloading part of the stress–strain curve, indenter tip radius and final indentation depth using the following formula:

$$Eff = \frac{dP}{{dh}}\frac{1}{{2\sqrt {R\left( {h_{t} + h_{r} } \right)} }}$$

where $\frac{dP}{{dh}}$ is the slope at maximum indentation, *R* is the radius of the spherical indenter tip, *h*_*t*_ and *h*_*r*_ represent maximum indentation depth and final contact depth (Fig. 1C,D,H). In this study, the fits were set as 100%, 100% and 65–85% in Hertzian model, JKR model and Oliver & Pharr model, respectively. Poisson’s ratio^41^ ν relates effective Young’s modulus (*Eff*) and Young’s modulus (*E*) by the following equation:

$$Eff = \frac{E}{{1 - v^{2} }}$$

Except *Eff*, in all models the Piuma software also enabled to directly calculate *E*, for which a Poisson’s ratio of 0.5 was pre-defined (for perfect incompressible materials). We determined both *Eff* and *E* for each biomaterial because the material property of tissues and the Poisson’s ratios are unknown.

### Stiffness of matrigen hydrogels and organs

**Figure 2:**
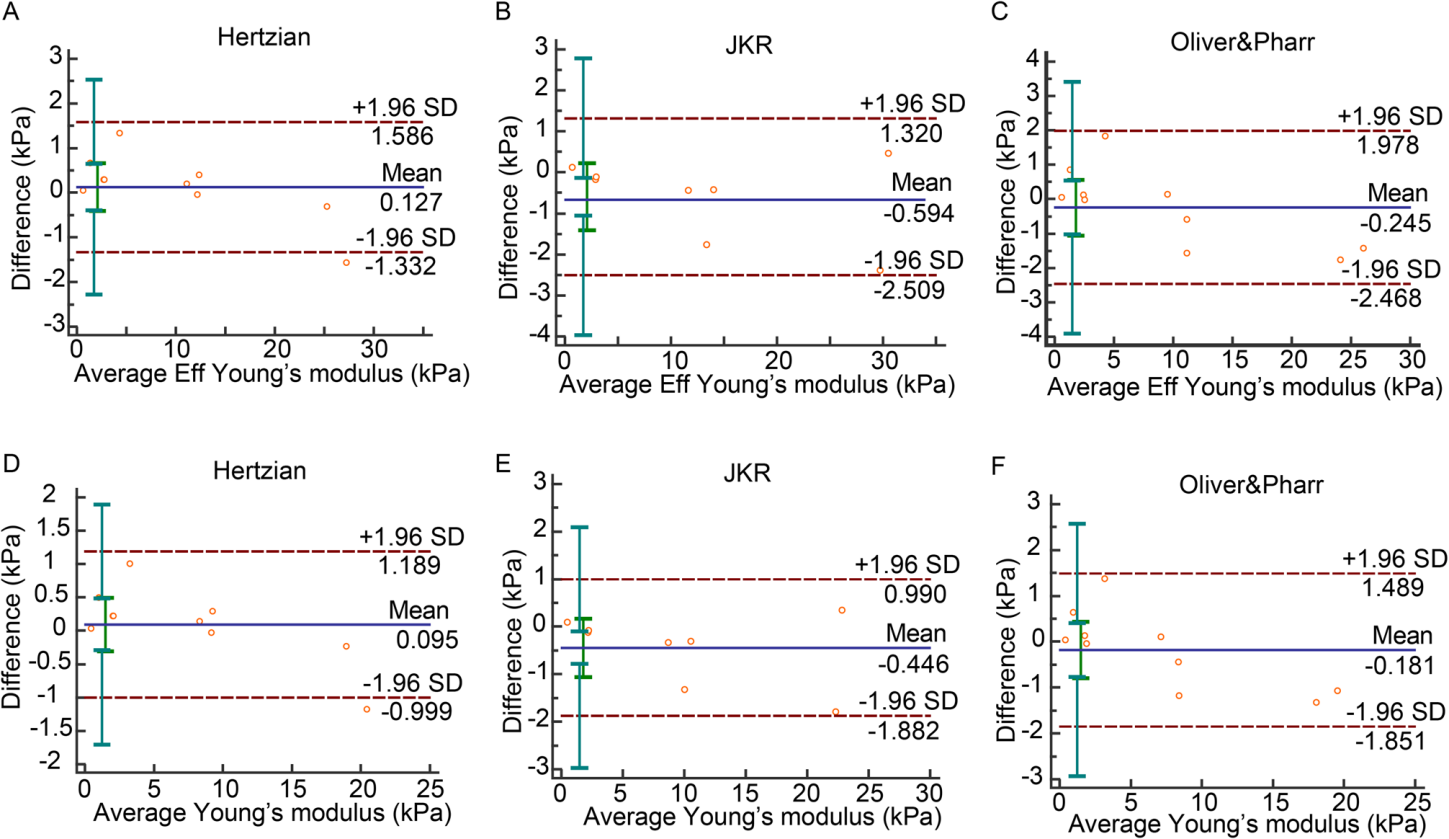
Bland–Altman plot of Matrigen hydogels. (**A**) Bland–Altman plot of *Eff* in Hertzian model. (**B**) Bland–Altman plot of *Eff* in JKR model. (**C**) Bland–Altman plot of *Eff* in Oliver & Pharr model. (**D**) Bland–Altman plot of *E* in Hertzian model. (**E**) Bland–Altman plot of *E* in JKR model. (F) Bland–Altman plot of *E* in Oliver & Pharr model.

Bland–Altman plots of Matrigen hydrogels showed mean stiffness differences in all measurements (Fig. 2). There was only one difference (spot) out of the 95% limits of agreement (− 1.96 SD to 1.96 SD) in the Hertzian model for both *Eff* and *E* (Fig. 2A,D). Differences in the other models were within the SD range (Fig. 2B,C,E,F). ICC values were larger than 0.8 and COVs were smaller than 15% in all models (Table 1), which demonstrates that all three models provide reliable results on gel specimen.

**Table 1:** Reliability of test–retest in Matrigen gels.

	Bland–Altman, 95% limits of agreement (kPa)	ICC, 95% CI	COV (%)
Eff in Hertzian	0.127 (− 1.332,1.586)	**0.9986 (0.9948,0.9997)**	**5.0666**
Eff in JKR	− 0.594 (− 2.509,1.320)	**0.9978 (0.9900,0.9996)**	**5.8171**
Eff in Oliver & Pharr	− 0.245 (− 2.468,1.978)	**0.9964 (0.9865, 0.9991)**	**8.3703**
E in Hertzian	0.095 (− 0.999,1.189)	**0.9986 (0.9948,0.9997)**	**5.0665**
E in JKR	− 0.446 (− 1.882,0.990)	**0.9978 (0.9900,0.9996)**	**5.8171**
E in Oliver & Pharr	− 0.181 (− 1.851,1.489)	**0.9964 (0.9865,0.9991)**	**8.3787**

Values that are in the range of good reliability are in bold.

**Figure 3:**
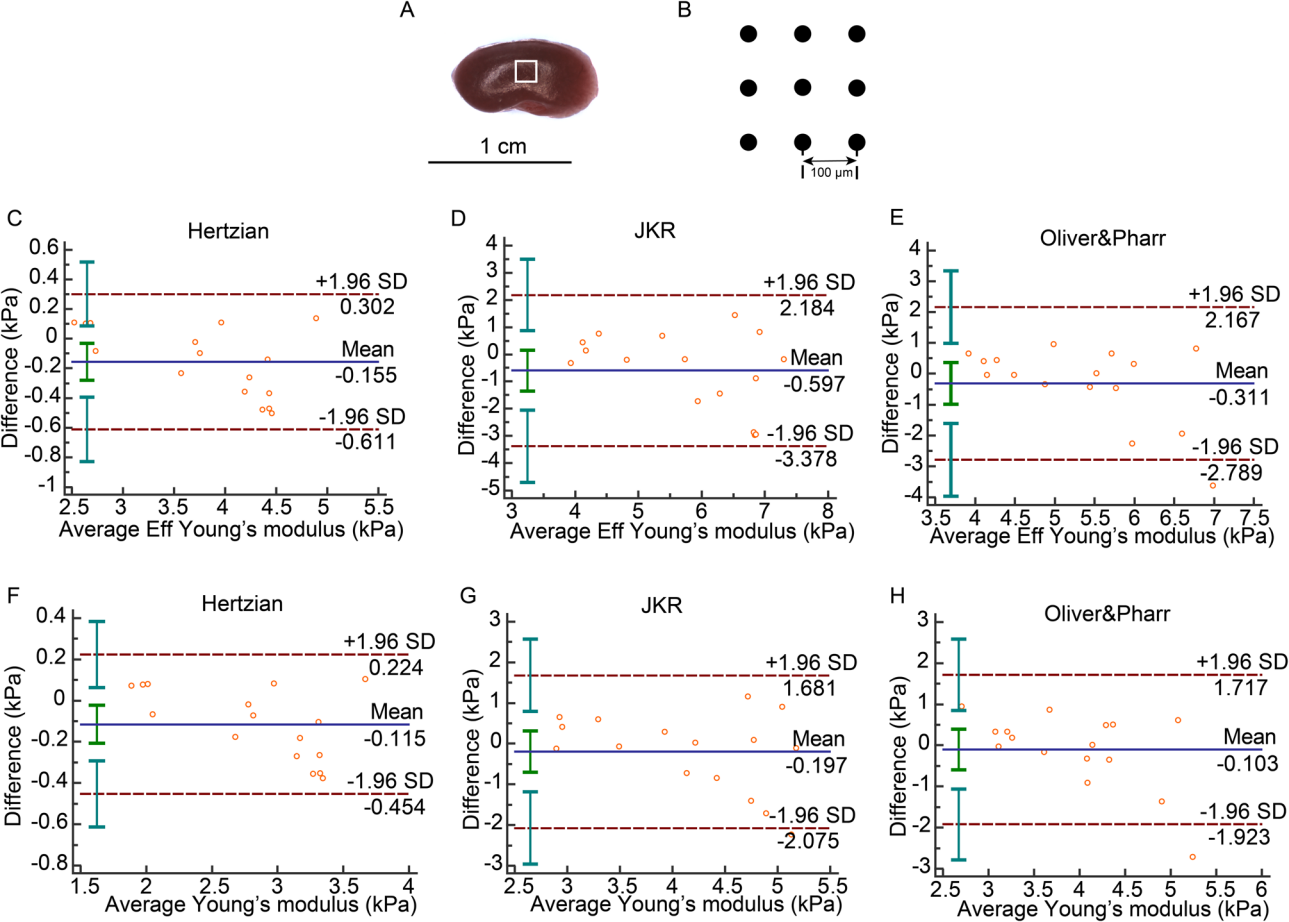
Renal indentation strategy and Bland–Altman plot. (**A**) Half of kidney was chopped from the middle line of side. White frame was the scan area on the tissue. (**B**) Sample was indented 9 times (3 × 3 matrix) in a 200 × 200 μm grid scan with 100 µm distance between measurements. (**C**) Bland–Altman of *Eff* in Hertzian model. (**D**) Bland–Altman plot of *Eff* in JKR model. (**E**) Bland–Altman plot of *Eff* in Oliver & Pharr model. (**F**) Bland–Altman plot of *E* in Hertzian model. (**G**) Bland–Altman plot of *E* in JKR model. (**H**) Bland–Altman plot of *E* in Oliver & Pharr model.

In the kidney, the Bland–Altman plots show that there was only one difference out of the 95% limits of agreement for *E* in JKR model, for *Eff* in the Oliver & Pharr model and for *E* in the Oliver & Pharr model (Fig. 3E,G,H). The differences in the other models were all within the SD range (Fig. 3C,D,F). Of note, the ICC value was larger than 0.8 and the COV was smaller than 15% only in the Hertzian model but not in the other models (Table 2).

**Table 2:** Reliability of test–retest in kidney.

	Bland–Altman, 95% limits of agreement (kPa)	ICC, 95% CI	COV (%)
Eff in Hertzian	− 0.155 (− 0.611,0.302)	**0.9686 (0.9124,0.9889)**	**5.0723**
Eff in JKR	− 0.597 (− 3.378,2.184)	0.5951 (− 0.1287,0.8572)	18.2387
Eff in Oliver & Pharr	− 0.311 (− 2.789,2.167)	0.5929 (− 0.1347,0.8565)	16.6833
E in Hertzian	− 0.115 (− 0.454,0.224)	**0.9693 (0.9143,0.9892)**	**5.0194**
E in JKR	− 0.197 (− 2.075,1.680)	0.6699 (0.0797,0.8836)	16.0643
E in Oliver & Pharr	− 0.103 (− 1.923,1.717)	0.6422 (0.0026,0.8738)	16.2013

Values that are in the range of good reliability are in bold.

**Figure 4:**
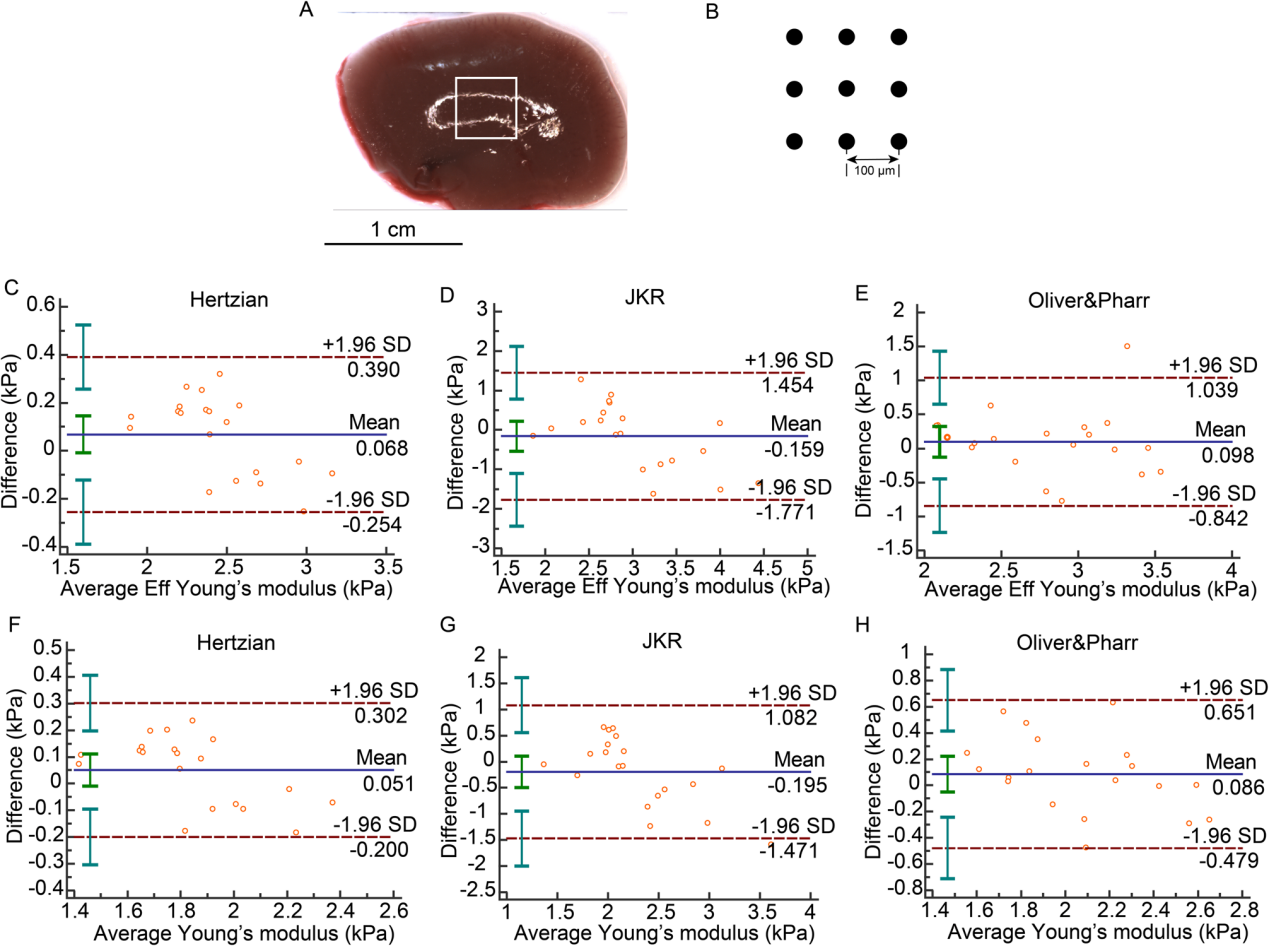
Hepatic indentation strategy and Bland–Altman plot. (**A**) A piece of liver was harvested from the left lobe. White frame was the scan area on the tissue. (**B**) Sample was indented 9 times (3 × 3 matrix) in a 200 × 200 μm grid scan with 100 µm distance between measurements. (**C**) Bland–Altman plot of *Eff* in Hertzian model. (**D**) Bland–Altman pot of Eff in JKR model. (**E**) Bland–Altman plot of *Eff* in Oliver & Pharr model. (**F**) Bland–Altman plot of *E* in Hertzian model. (**G**) Bland–Altman plot of *E* in JKR model. (**H**) Bland–Altman plot of *E* in Oliver & Pharr model.

In the liver, the Bland–Altman plots uncovered mean stiffness differences between test and retest data. Except for *Eff* in the Hertzian model and *E* in the JKR model, there were no differences out of the 95% limits of agreement (Fig. 4C-H). The ICC values of *E* and *Eff* exceeded 0.8 consistently only in the Hertzian model but not Oliver & Pharr and JKR models. The COV values were smaller than 15% in the Hertzian and Oliver & Pharr models but not in the JKR model (Table 3).

**Table 3:** Reliability of test–retest in liver.

	Bland–Altman, 95% limits of agreement (kPa)	ICC, 95% CI	COV (%)
Eff in Hertzian	0.679 (− 0.254,0.390)	**0.9303 (0.8269,0.9722)**	**5.0091**
Eff in JKR	− 0.159 (− 1.771,1.454)	0.6240 (0.0667,0.8501)	19.1874
Eff in Oliver & Pharr	0.098 (− 0.842,1.039)	0.7507 (0.3811,0.9006)	**12.0356**
E in Hertzian	0.051 (− 0.200,0.302)	**0.9251 (0.8142,0.9702)**	**5.1724**
E in JKR	− 0.195 (− 1.472,1.082)	0.6078 (0.0265,0.8437)	20.5038
E in Oliver & Pharr	0.086 (− 0.479,0.651)	**0.8048 (0.5155,0.9222)**	**10.0323**

Values that are in the range of good reliability are in bold.

**Figure 5:**
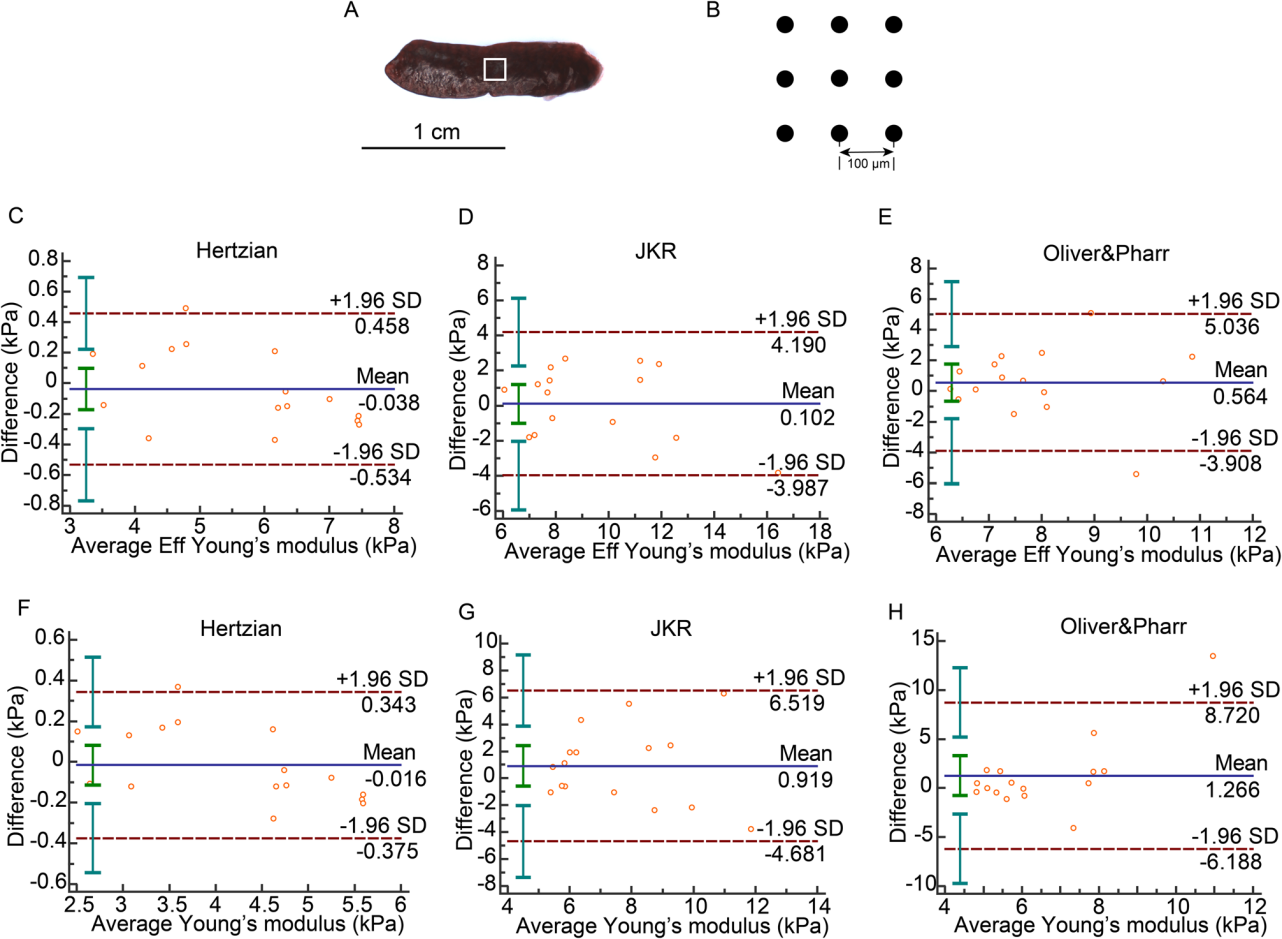
Splenic indentation strategy and Bland–Altman plot. (**A**) An intact spleen of mouse. White frame was the scan area on the tissue. (**B**) Sample was indented 9 times (3 × 3 matrix) in a 200 × 200 μm grid scan with 100 µm distance between measurements. (**C**) Bland–Altman plot of *Eff* in Hertzian model. (**D**) Bland–Altman of *Eff* in JKR model. (**E**) Bland–Altman plot of *Eff* in Oliver & Pharr model. (**F**) Bland–Altman plot of *E* in Hertzian model. (**G**) Bland–Altman plot of *E* in JKR model. (**H**) Bland–Altman plot of *E* in Oliver & Pharr model.

In the spleen, the Bland–Altman plots showed that the average stiffness differences of test–retest data were within the SD range in the JKR model. There was only one difference result out of the scope in the Hertzian and Oliver & Pharr models (Fig. 5C-H). The ICC values of *E* and *Eff* were consistently larger than 0.8 in the Hertzian and JKR models but not in the Oliver & Pharr model. The COVs of the *E* and *Eff* values were smaller than 15% in Hertzian but not in the other models (Table 4).

**Table 4:** Reliability of test–retest in spleen.

	Bland–Altman, 95% limits of agreement (kPa)	ICC, 95% CI	COV (%)
Eff in Hertzian	− 0.038 (− 0.534,0.458)	**0.9924 (0.9787,0.9973)**	**3.1172**
Eff in JKR	0.102 (− 3.987,4.190)	**0.8675 (0.6307,0.9533)**	15.0115
Eff in Oliver & Pharr	0.564 (− 3.908,5.036)	0.3334 (− 0.8583,0.7649)	20.3557
E in Hertzian	− 0.016 (− 0.375,0.343)	**0.9931 (0.9807,0.9976)**	**2.9890**
E in JKR	0.919 (− 4.681,6.519)	0.5113 (− 0.3622,0.8277)	27.1063
E in Oliver & Pharr	1.266 (− 6.188,8.720)	− 0.3404 (− 2.7364,0.5274)	42.3569

Values that are in the range of good reliability are in bold.

**Figure 6:**
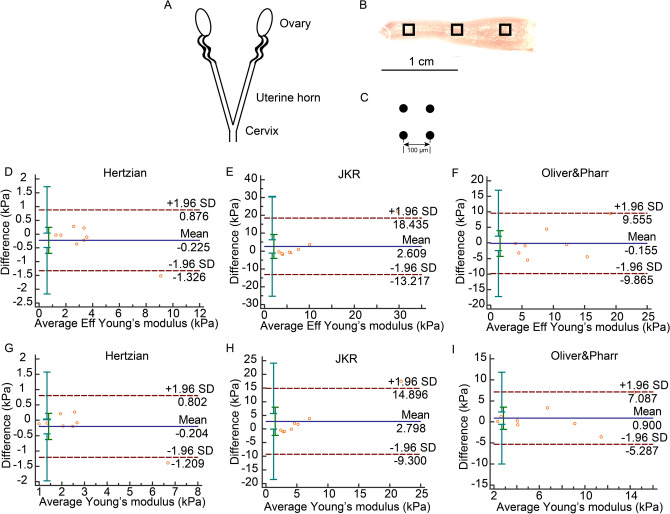
Uterine indentation strategy and Bland–Altman plot. (**A**) Schematic diagram of mouse whole uterus. (**B**) Opened left uterus horn. Black frames were three scan areas located on proximal, middle and distal parts, respectively. (**C**) In each scan, there were four indentation spots with 100 μm distance between measurements in a 100 × 100 μm square. (**D**) Bland–Altman plot of *Eff* in Hertzian model. (**E**) Bland–Altman plot of *Eff* in JKR model. (**F**) Bland–Altman plot of *Eff* in Oliver & Pharr model. (**G**) Bland–Altman plot of *E* in Hertzian model. (**H**) Bland–Altman plot of *E* in JKR model. (**I**) Bland–Altman plot of *E* in Oliver & Pharr model.

In uterus, Bland–Altman plots revealed only one difference out of the 95% limits of agreement between indentations in both the Hertzian and JKR models (Fig. 6D,E,G,H) but not in Oliver & Pharr model (Fig. 6F,I). ICC values were larger than 0.8 in Hertzian and Oliver & Pharr models but not JKR model. In contrast, COVs were smaller than 15% only in Hertzian model but not other models (Table 5).

**Table 5:** Reliability of test–retest in uterus.

	Bland–Altman, 95% limits of agreement (kPa)	ICC, 95% CI	COV (%)
Eff in Hertzian	− 0.225 (− 1.326,0.876)	**0.9861 (0.9371,0.9972)**	**11.6893**
Eff in JKR	2.609 (− 13.217,18.435)	0.7926 (0.0607,0.9577)	64.4623
Eff in Oliver & Pharr	− 0.155 (− 9.865,9.555)	**0.8295 (0.2278,0.9652)**	34.7149
E in Hertzian	− 0.204 (− 1.209,0.802)	**0.9780 (0.9002,0.9955)**	**14.1841**
E in JKR	2.798 (− 9.300,14.896)	0.7512 (− 0.1267,0.9492)	71.0078
E in Oliver & Pharr	0.900 (− 5.287,7.087)	**0.8778 (0.4465,0.9751)**	31.8501

Values that are in the range of good reliability are in bold.

## Discussion

Piuma nanoindentation technology has been widely used in research on biomaterial stiffness of hard animal organs, for example in bone^25^, in ear, ala nasi, and septum on both the cellular and the extracellular matrix (ECM) levels^24,26^, in the knee joint^22^, in articular cartilage^23^. Other examples are human donor cornea^27^, fibrotic intestinal tissue^30^, pancreatic acellular scaffolds^31^, soft plates^28^ and particularly calcified aneurysmal abdominal aortas^29^. The feasibility and reliability of this technology in measuring the stiffness of soft biological materials, particularly organs ex vivo, is unknown. Compared with hard biomaterials, certain properties of soft biomaterials, such as viscoelasticity and adhesion, are more prone to deviations in nanoindentation. Our study is the first to use this technology to test the stiffness of soft biological organs ex vivo, particularly from mice, which are widely used for modeling human and animal diseases. We applied the Piuma nanoindentation technology, which is easy to use and utilizes a specific probe to measure Young’s Moduli to match with the specific sample properties of the tissue^42–44^. Different tissue contains different mechanical properties, therefore, different protocols should be applied in tissues with some special characterizations^45^. Since there is no way to judge the feature of tested samples before an experiment, we analyzed both the loading and unloading parts for their elastic behavior detection. In addition to the operation of the system and the development of measurement strategies, the preparation and fixation of tissue is expected to be important. An irregular tissue is impossible to be tested as the device only recognizes flat and stable surfaces, and the calculation of stiffness would be affected by the condition of sample. For example, if the tested surface is a slope (Supplement 1, Fig 1A), the contacted area would not be fully indented by the tip of probe, which means the losing depth and force could led to a mistake in stiffness measurement. A globose organ (Supplement 1 Fig 1B) is also untestable because it cannot be stabilized during the process of measurement. Furthermore, it is impossible for this technology to test a sunk surface (Supplement 1 Fig 1C) due to the obstacle at the edge of the tissue. A lumpy surface (Supplement 1 Fig 1D) would not only affect the accuracy of the measurement results but also cause the probe cantilever to be damaged due to jamming. Together, the tested tissue needs to be prepared testable in an appropriate shape and size. We did overcome these possible limitations for the feasibility and reliability of the nanoindentation technology to measure soft organ stiffness by using isolated kidneys, liver, spleen and uterus dissected in an appropriate manner. We confirmed the feasibility and reliability of the results by a comparative study of this technology to Matrigen hydrogels. Matrigen hydrogels at a given stiffness, stable shape, appropriate thickness and flat surface were taken as a quality control, although their given stiffness is not considered golden-standard. Nevertheless, our Bland–Altman plots, ICCs and COVs demonstrated a good reliability of the gels used. Therefore, we conclude that nanoindentation technology works well and is reliable in our laboratory settings and on this material.

We next tested the technology in measuring the stiffness of four organs ex vivo, namely the kidney, liver, spleen and uterus. Although the Bland–Altman plots did not give us numerous out-of-qualification results, the reliability of the results from the four organs hardness in different models can only be validated by comparing the ICCs and COVs. In the four organs, all Hertzian model’s results followed the quantified criteria of ICCs and COVs showed reliable results. The results in JKR or Oliver & Pharr models did not always meet the high quality criteria. The reason for the observed differences may rely on differences between specimens in stickiness under unloading state.

For example, in the JKR model, even on the same sample, some spots are sticky, while some single indentations show no adhesion, as shown in Fig. 1E, which would increase the variation between test and retest. Thus, our results show that the hardness of the four organs under study is best calculated using the Hertzian model under forced indentation. Of note, this model has been used by others researchers who utilized the nanoindentation technology in their studies^27–29^, while other studies did not report the model used^30,31^. In addition, comparing the results of the uterus with the three other organs, we found that even in the Hertzian mode, the COV value of the uterus is 11.6893% in the case of calculating the hardness according to *Eff* and 14.1841% in the case of calculating the stiffness according to *E*, which were very close to the threshold and much higher than the COV values of the other three organs in the Hertzian model. This indicates that the variability between repeated measurements of the uterus is greater than that of the liver, kidney and spleen. A possible reason is that the uterus is smaller and thinner than the other three organs, and in the course of the experiment, we found that the edge of the smaller and thinner organ uterus was more likely to be rolled up causing a similar situation shown in Supplement 1 Fig 1B, which is expected to affect the results of the measurements. Therefore, the reliability of the method is better in relatively large and thick soft organs ex vivo.

Moreover, the successful application of nanoindentation is highly depended on material features such as the shape of the tissue being measured; measurement of biological materials with complicated rough surface tends to be difficult. When a tissue is manually transformed into a material that can be tested, it is unknown that whether its elasticity keeps the same property as the elasticity of the original organ, and whether part of the elasticity of the organ can represent its overall elasticity. Accordingly, for certain organ studies, in vivo testing may be a better or sometimes even the only option to provide detailed insights into the organ’s mechanical properties. However, in vivo tests are likely interfered and affected by other factors during the measurement process, so perhaps it is an advantage that the nanoindenter can be directly contacted with the target material for measurement. At present, there are more limitations and shortcomings in the application of this technology. For example, its validity and authenticity still need to be further verified, and the standardized routine for biomaterial nanoindentation has not yet been established. Therefore, we cannot be certainly sure whether it will become an indispensable tool in the research of mechanical biology and biomechanics of soft organs and tissues. However, as the research and development of this technology is going more and more in-depth, we expect it will have great opportunities to be applied to research in multiple fields such as physiology and pathology of soft organs.

## Conclusion

Piuma nanoindentation technology is an easy and feasible method to test the stiffness of ex vivo organs, such as kidney, liver, spleen and uterus. In small and thin tissues with disorder surface, we expect that the variation of results will increase. The Hertzian model is the most reliable method to measure the passive mechanical property of soft organs and biomaterials ex vivo. JKR and Oliver & Pharr models did not thoroughly provide reliable results.

## Materials and methods

### Animals

Kidneys, liver and spleen were dissected from 5 week-, 10 week-, 20 week- and 30 week-old C57BL/6 N mice. For kidneys and spleen, 2 mice of each sex and age were used. For liver, 2 to 4 mice of each sex and age were used. For uterus, eight mice were used, all these mice were around 100 days old. The experiments were approved by the regulations of local animal care committee (LAGeSo, Berlin, Germany) and the animal welfare officers of the Max Delbrück Center for Molecular Medicine (MDC) (No. X 9011/19).

### Matrigen hydrogels

Hydrogels with different stiffness (1 kPa, 2 kPa, 4 kPa, 8 kPa, 12 kPa and 25 kPa) were purchased from Softwell, Matrigen, Matrigen Life Technologies, Brea, CA and used for quality control (1 kPa, 2 kPa, 4 kPa, 8 kPa, 12 kPa and 25 kPa; N = 1 to 3).

### Preparation of tissues

Left and right kidneys were respectively divided into two from the middle line of side **(**Fig. 3A**)**, the four parts were all taken into indentation. Liver samples were taken from left lobe (Fig. 4A). Spleens were whole harvested for usage in the experiments (Fig. 5A). Left uterus horn was selected and opened (Fig. 6A,B). All organs were cleaned with removal of visible blood, fat, membrane or vessels on the surface of organs, but avoiding damage the parenchyma of them. To obtain a flat surface, we pasted all samples to the bottom of 4 cm diameter petri-dishes with Shellac (Sigma) so that the outer surface was leveled. Tissue samples were immersed in PBS (NaCl 0.137 M, KCl 0.0027 M, Na_2_HPO_4_ 0.01 M, KH_2_PO_4_ 0.0018 M; pH 7.4).

### Nanoindentation

To determine elastic properties, we used a displacement-controlled nanoindenter instrument (Piuma; Optics11, Amsterdam, The Netherlands). The device utilizes a ferrule-top cantilever probe^32,33^ to apply load and simultaneously measure indentation depth using a fiber optic based readout (Fig. 1A). We used a spherical probe with a radius of 50 µm and a cantilever stiffness of 0.5 N/m. Cantilever bending calibrations were performed before each series of experiments by indenting a rigid surface and equating cantilever bending to probe displacement. Afterwards, the probe was focused on an appropriate area on tissue surface (Figs. 1B, 3A, 4A, 5A, 6B). Each gel was indented 25 times (5 × 5 matrix) in an 800 × 800 μm grid scan with 200 µm distance between measurements. Kidney, liver and spleen samples were indented with 9 indentations (3 × 3 matrix) in a 200 × 200 μm grid scan (Figs. 3B, 4B, 5B). In uterus, three indentation matrixes with 4 single indentations in 100 × 100 μm grid were tested in proximal, middle and distal parts of uterus, respectively (Fig. 6B,C). The applied indentation protocol was composed of a loading phase for 4 s at 8000 nm indentation depth, which was held for one second, and then an unloading phase for 4 s. All scans were done twice for the analysis of reliability. The average of all the results in the four sections from left and right kidneys was presented as renal elasticity. The stiffness of gel, liver and spleen was expressed as the mean value of all results in each scan. Three scans results’ average was taken as uterine hardness. All single indentation values were calculated by Piuma Dataviewer version 2.2 (Piuma; Optics11, Amsterdam, The Netherlands).

### Bland–Altman plots and coefficients

Bland–Altman plots (a graphical method to plot the difference scores of two measurements against the mean for each subject)^34,35^, intraclass correlation coefficients (ICCs) and within-subject coefficient of variations (COVs) were used to analyze the reliability of test–retest results. If the difference value of test–retest results is between 95% limits of agreement in the Bland–Altman plots, it means the reliability is good. If the value of ICC is greater than 0.8, there is good reliability between the measurement and re-measurements. If COVs are smaller than 15%, it is considerable that the test–retest result is reliable.

All analyses were performed using SPSS 19.0 (Chicago, USA), GraphPad Prism 7.0 (San Diego, USA) or MedCalc 19.3 software (Belgium).

### Ethics approval and consent to participate

The usage of mice was abided by the regulations of local animal care committee (LAGeSo, Berlin, Germany) and the animal welfare officers of the Max Delbrück Center for Molecular Medicine (MDC) (No. X 9011/16). There are no ethical concerns.

## Supplementary information

Supplementary Information 1.

Supplementary Information 2.

## Data Availability

The data and protocol can be obtained by contacting Michael Gotthardt.

## Abbreviations

*Eff*: Effective Young’s modulus
*E*: Young’s modulus
ICC: Intraclass Correlation Coefficient
CI: Confidence Interval
COV: Within-subject coefficient of variation
