# Author Correction: Assessment of nanoindentation in stiffness measurement of soft biomaterials: kidney, liver, spleen and uterus

**DOI:** 10.1038/s41598-021-86044-1

**Published:** 2021-03-23

**Authors:** Guanlin Wu, Michael Gotthardt, Maik Gollasch

**Affiliations:** 1 Max Delbrück Center for Molecular Medicine (MDC) in the Helmholtz Association, Robert‑Rössle‑Straße 10, 13125 Berlin, Germany; 2 Experimental and Clinical Research Center (ECRC), Charité–Universitätsmedizin Berlin, Berlin, Germany; 3 German Center for Cardiovascular Research (DZHK), Partner Site Berlin, Berlin, Germany; 4 Department of Internal and Geriatric Medicine, University of Greifswald, University District Hospital Wolgast, Greifswald, Germany; 5 Medical Clinic of Nephrology and Internal Intensive Care, Charité-Universitätsmedizin Berlin, Berlin, Germany

Correction to: *Scientific Reports* https://doi.org/10.1038/s41598-020-75738-7, published online 02 November 2020

The original version of this Article contained errors.

In the original version of this Article an incorrect email address for Guanlin Wu was quoted and Maik Gollasch was omitted as a corresponding author. Correspondence and request for materials should be addressed to wuguanlin105109@gmail.com and maik.gollasch@charite.de.

In addition, Michael Gotthardt was incorrectly affiliated with ‘Medical Clinic of Nephrology and Internal Intensive Care, Charité-Universitätsmedizin Berlin, Berlin, Germany’. The correct affiliations are listed below.

Max Delbrück Center for Molecular Medicine (MDC) in the Helmholtz Association, Robert‑Rössle‑Straße 10, 13125 Berlin, Germany.

German Center for Cardiovascular Research (DZHK), Partner Site Berlin, Berlin, Germany.

Finally, Maik Gollasch was incorrectly affiliated with ‘German Center for Cardiovascular Research (DZHK), Partner Site Berlin, Berlin, Germany’. The correct affiliations are listed below.

Experimental and Clinical Research Center (ECRC), Charité–Universitätsmedizin Berlin, Berlin, Germany.

Department of Internal and Geriatric Medicine, University of Greifswald, University District Hospital Wolgast, Greifswald, Germany.

Medical Clinic of Nephrology and Internal Intensive Care, Charité-Universitätsmedizin Berlin, Berlin, Germany.

These errors have now been corrected in the PDF and HTML versions of the Article.

