# Supplementary material for: Assessment of nanoindentation in stiffness measurement of soft biomaterials: kidney, liver, spleen and uterus
Source: Sci Rep. 2020 Nov 2;10:18784. doi: 10.1038/s41598-020-75738-7 (PMC7606463; doi:10.1038/s41598-020-75738-7)
Supplement: Supplementary file 1 — Supplementary Information 1. [file 41598_2020_75738_MOESM1_ESM.docx]

**Supplement 1 Fig 1.** Inappropriate tissues for indentation. (A) Tissue with gradient surface. (B) Unstable tissue. (C) Tissue with sunk surface. (D) Tissue with lumpy surface.
